# A Sensor Dynamic Measurement Error Prediction Model Based on NAPSO-SVM

**DOI:** 10.3390/s18010233

**Published:** 2018-01-15

**Authors:** Minlan Jiang, Lan Jiang, Dingde Jiang, Fei Li, Houbing Song

**Affiliations:** 1 College of Mathematics, Physics and Information Engineering, Zhejiang Normal University, Jinhua 321004, China; sa1105587@163.com (L.J.); m18329019792@163.com (F.L.); 2 School of Astronautics and Aeronautic, University of Electronic Science and Technology of China, Chengdu 611731, China; 3 Department of Electrical, Computer, Software, and Systems Engineering, Embry-Riddle Aeronautical University, Daytona Beach, FL 32114, USA; h.song@ieee.org

**Keywords:** sensors, dynamic measurement errors, prediction, improved PSO, support vector machine

## Abstract

Dynamic measurement error correction is an effective way to improve sensor precision. Dynamic measurement error prediction is an important part of error correction, and support vector machine (SVM) is often used for predicting the dynamic measurement errors of sensors. Traditionally, the SVM parameters were always set manually, which cannot ensure the model’s performance. In this paper, a SVM method based on an improved particle swarm optimization (NAPSO) is proposed to predict the dynamic measurement errors of sensors. Natural selection and simulated annealing are added in the PSO to raise the ability to avoid local optima. To verify the performance of NAPSO-SVM, three types of algorithms are selected to optimize the SVM’s parameters: the particle swarm optimization algorithm (PSO), the improved PSO optimization algorithm (NAPSO), and the glowworm swarm optimization (GSO). The dynamic measurement error data of two sensors are applied as the test data. The root mean squared error and mean absolute percentage error are employed to evaluate the prediction models’ performances. The experimental results show that among the three tested algorithms the NAPSO-SVM method has a better prediction precision and a less prediction errors, and it is an effective method for predicting the dynamic measurement errors of sensors.

## 1. Introduction

Today, sensors are widely used in the real world [1,2,3,4], and sensor errors are one of the keys to evaluate the measurement quality of the sensor results. With the development of modern measurement technology, dynamic measurements have gradually become the mainstream of modern measurement techniques. 

As an effective way to improve measurement accuracy and reduce measurement errors, real-time error correction of sensors has been widely used in dynamic measurements. Predicting the dynamic measurement error is useful to correct the errors of sensors. Dynamic measurement errors of sensors are difficult to model with traditional mathematics because they have four features [5]: time-varying, randomness, correlation and dynamic. Because of its complexity, predicting the dynamic errors has been a popular research field [6,7,8,9].

In recent years, several modeling methods were used to predict dynamic errors like the gray theory, Bayesian networks and neural network. Every method has its own advantages and drawbacks. The harmonic analysis method is suitable for modeling periodic sequences, but it is not suitable for random sequences [10]. Bayesian networks is a prediction modeling method, however, it requires the prior distribution and independent samples, which is difficult to achieve in real systems [11]. Grey theory model can be constructed by a few samples, but it only depicts a monotonically increasing or decreasing process [12,13]. Artificial neural network has a good non-linear mapping performance, however, it has disadvantages, such as over-fitting and easily falling into a local minimum [14,15,16]. 

Support vector machine (SVM) adopts structural risk minimization to improve its generalization ability [17]. It can better solve the problems of nonlinear data and small samples. SVM has been widely applied to solve function fitting problems. However, the generalization ability of SVM depends heavily on the appropriate parameters, and the model’s parameters have a huge influence on the precision of the model predictions [18]. Thus, many optimization algorithms have been adopted to optimize the SVM parameters [19,20,21], like the particle swarm optimization (PSO) algorithm, genetic algorithm (GA) and glowworm swarm optimization (GSO) algorithm. These methods have limitations, as the particle swarm optimization and genetic algorithm fall into local extremes easily [22], and the glowworm swarm optimization algorithm has low convergence precision and slow convergence speed [23]. The NAPSO algorithm is an improved particle swarm optimization algorithm based on the natural selection strategy and simulated annealing mechanism. These two methods are used to improve the global search ability and convergence speed. In this study, a method of dynamic measurement error prediction for sensors based on NAPSO optimize support vector machine is proposed.

The rest of the paper is organized as follows: in Section 2, a detailed overview of the SVM algorithm is provided. Then, in Section 3, the PSO and NAPSO algorithms and the process of optimization are described briefly. Section 4 reports on a simulation of the dynamic measurement error prediction model. The results of experiments are discussed in Section 5. Conclusions are drawn in the last section. 

## 2. SVM Algorithm 

### 2.1. SVM

SVM is a machine learning method based on the statistical learning theory developed in mid-1990s. The basic idea of SVM is that the data of input space *R^n^* are mapped to a high dimensional feature space *F* by a nonlinear mapping, then the linear regression operations are finished in the high dimensional feature space [24].

For a given training dataset {(*x_i_*,*y_i_*), *i* = 1,2,…,*n*}, *x_i_* is a n-dimensional input vector and *y_i_* is the corresponding output value, *φ*(*x*) is the nonlinear mapping from input space *R^n^* to high dimensional feature space *F*.

(1) $$R^{n}\to F:x\to \varphi (x)$$

The regression function of SVM is formulated as follows:

(2) $$f(x)=[\omega \times \varphi (x)]+b\;\omega \in R^{m},b\in R$$

where *ω* is the weight vector and *b* is the threshold, the main goal of the SVM is to find the optimal *ω*, the weight vector *ω* can be expressed by a linear combination of the training examples. Thus, the Equation (2) takes the form:

(3) $$f(x)=\sum_{i=1}^{n}\alpha_{i}x_{i}{}^{T}x+b\;b\in R$$

where *α_i_* are Lagrangian multipliers. In the feature space, Equation (4) can be expressed as follows:

(4) $$f(x)=\sum_{i=1}^{n}\alpha_{i}\varphi (x_{i}{}^{T})\varphi (x_{j})+b\;b\in R$$

Introduce the Kernel function *K*(*x_i_*,*y_j_*):

(5) $$K(x_{i},x_{j})=\varphi (x_{i}{}^{T})\varphi (x_{j})$$

Finnally, the SVM regression function is formulated as:

(6) $$f(x)=\sum_{i=1}^{n}\alpha_{i}K(x_{i},x_{j})+b$$

### 2.2. Kernel Function

Kernel function is a key concept of SVM, the performance of SVM mainly depends on the kernel function. As shown in the Equation (1), the kernel function establishes a relation between the input space *R^n^* and the high dimensional feature space *F*. Different selection of kernel functions will construct different regression models:

(7) $$K(x_{i},x_{j})=\phi {\left(x_{i}\right)}^{T}\times \phi (x_{j})$$

The common kernel functions include the polynomial kernel function, linear kernel function, fourier kernel function and radial basis function (RBF) kernel function. The kernel function parameters has a directly influence on the complexity of the function, RBF kernel function has the advantages of fewer parameters and good performance. Thus, RBF kernel function is used in this paper. 

The RBF kernel function is expressed as follows:

(8) $$K(x_{i},x_{j})=\exp\left\{-\frac{{\left|x_{i}-x_{j}\right|}^{2}}{2\sigma^{2}}\right\}$$

where *σ* is the width coefficient of the kernel function.

The SVM parameters determine both its generalization ability and learning ability, the punishment coefficient *C* and RBF kernel function width *σ* have a direct impact on the accuracy and efficiency of the SVM prediction model. *C* adjusts the balance between generalization and empirical error. When *C* is greater, the model’s complexity will be increased and it will fall into the “over-fitting” phenomenon easily, but if *C* is too small, the model’s complexity will be reduced and it will fall into the “under-fitting” phenomenon easily. The value of *σ* affects the complexity of the sample data distribution in feature space. In this paper, NAPSO algorithm is used to optimize the two parameters to achieve better prediction results.

## 3. SVM Parameters Optimization Based on NAPSO

### 3.1. PSO

Particle swarm optimization was proposed by Eberhart and Kennedy in 1995 [25], PSO was derived from research on bird flocks’ preying behavior. When a flock of birds is looking for food in an area randomly, if there is only one piece of food in the area being searched, the most effective and simple method to find the food is to follow the bird that is closest to the food.

In a PSO algorithm, every single solution is a particle in the search space. Each particle has a fitness value, which is determined by an optimization function, each particle has its own velocity and position. The velocity and position of each particle will be changed by the particle best position and global best position. The update equations of the velocity and position are shown by the following expression:

(9) $$v_{i.d}(t+1)=\omega v_{i.d}(t)+c_{1}r_{1}[p_{best}-x_{i.d}(t))]+c_{2}r_{2}[g_{best}-x_{i.d}(t))]$$

(10) $$x_{i.d}(t+1)=x_{i.d}(t)+v_{i.d}(t+1)$$

In the d-dimensional space, *t* is the iteration number, *υ_i,d_*(*t*) is the velocity of particle *i* at iteration *t*, *υ_i,d_*(*t* + 1) is the velocity of particle *i* at iteration *t* + 1, *x_i,d_*(*t*) is the position of particle *i* at iteration *t*, *x_i,d_*(*t* + 1) is the position of particle *t* at iteration *t* + 1, *ω* is the inertia weight. *c*_1_ is the cognition learning factor, *c*_2_ is the social learning factor, *r*_1_ and *r*_2_ are random numbers that are uniformly distributed in [0,1], *p_best_* is the particle best position for the individual variable of particle *i*, *g_best_* is the global best position variable of the particle swarm.

The initial position and velocity of each particle are randomly generated and will be updated based on the Equations (9) and (10) until a satisfactory solution is found. In the PSO algorithm, a single particle moves to its *p_best_* and *g_best_*, each particle’s movement generates a fast convergence, thus the PSO algorithm converges rapidly. However, the fast convergence also makes the update of each particle depend too much on its *p_best_* and *g_best_*, which makes the algorithm fall into local optima and easily converge prematurely. Therefore, in this paper, an improved PSO algorithm (NAPSO) is used to optimize the parameters of SVM.

### 3.2. NAPSO

The NAPSO algorithm is an improved PSO algorithm based on the methods of natural selection and simulated annealing. In the NAPSO algorithm, the simulated annealing mechanism is used to improve the ability of the algorithm to jump out of a local optimum trap, while the natural selection method is employed to accelerate the rate of convergence.

The NAPSO algorithm starts with a set of random velocities and positions. Before the iteration, each particle’s personal best position and global best position are calculated by the fitness function. Each particles update its velocity and position by Equations (9) and (10) at each iteration.

After updating a particle’s speed, position *l* and fitness value *f*′, the particle moves to a random position $l_{1}^{\prime }$ in its neighborhood and computes its new fitness value $f_{1}^{\prime }$. The movement formula is expressed as follows:

(11) $$l_{1}^{\prime }=l+r_{3}\times [v_{max}-v_{min}]\times r_{1}$$

where *r*_3_ is the normally distribution random numbers of d-dimension that are distributed in [0,1], *υ_max_* is the maximum value of the velocity, and *υ_min_* is the minimum value of the velocity.

When $f_{1}^{\prime }>g_{best}$, we keep the position *l*. When $f_{1}^{\prime }<g_{best}$, if $f_{1}^{\prime }>f^{\prime }$, use the new position $l_{1}^{\prime }$ to replace the position *l* by the simulated annealing operation, where the operation of simulated annealing is expressed as follows:

(12) $$l=\left\{\begin{array}{cc} l & if\;\exp((-1)\times (f_{1}^{\prime }-f^{\prime })/T>r_{4})\\ l_{1}^{\prime } & if\;\exp((-1)\times (f_{1}^{\prime }-f^{\prime })/T<=r_{4}) \end{array}\right\}$$

where *r*_4_ is a random number that is uniformly distributed in [0,1], *T* is the simulated annealing temperature.

Each particle uses the simulated annealing operation to determine whether to accept the new position, and then updates the particle’s *p_best_* and *g_best_* by its position. The simulated annealing operation can significantly enhance the ability of the algorithm to jump out of the local optimum trap. At the end of each iteration, all particles have been ranked by their fitness values, from best to worst, and using the better half to replace the other half. In this way, the stronger adaptability particles are saved. Finally, the NAPSO algorithm is terminated by the satisfaction of a termination criterion. 

The pseudo code of the NAPSO algorithm is presented as the Algorithm 1.
**Algorithm 1:** NAPSO **Input**
*ω*, *c*_1_, *c*_2_, *T***Output**
*g_best_* **Initialization:**
*x*, *p_best_*, *g_best_* **while**
*t* < maximum number of iterations and *g_best_* > minimum fitness **do**  **for** each particle **do**    update the velocity *v*, position l, and fitness *f*′    find a new position $l_{1}^{\prime }$ in the neighborhood and calculate its fitness value $f_{1}^{\prime }$    **if1** ($f_{1}^{\prime }$ < *g_best_*) **then**      **if2** ($f_{1}^{\prime }-f^{\prime }<0$) **then**        accept the new position $l_{1}^{\prime }$      **else if2**        accept the new position $l_{1}^{\prime }$ by the simulated annealing operation      **end if2**    **else if1**      accept the old position *l*    **end if1**    update the *p_best_*, *g_best_* and Simulated temperature *T*  **end for**  rank all particles by their fitness value, use the better half to replace the other half.  *t* = *t* + 1 **end while** return the *g_best_*

The simulated annealing operation will slow the rate of convergence, thus increasing the convergence time. The natural selection operation will reduce the diversity of samples. However, these two operations can compensate for each other, as the simulated annealing operation can increase sample diversity, and the natural selection operation can speed up the convergence rate. These two operations are used to both ensure the convergence rate of the algorithm and guarantee that the ability of the algorithm to jump out of the local optimal trap can be enhanced.

### 3.3. Optimization Process

The NAPSO algorithm is applied to optimize the SVM parameters *C* and *σ* as follows:

Step 1: Initialize the NAPSO algorithm, set the number of particles velocity, particles positions and the other parameters. Because the search space is 2-dimensional, the position of each particle contains two variables. Set *T* to be the simulated temperature; the initial *T* is 5000 °C, and the lower limit of *T* is 1 °C. Calculate the fitness value of each particle. The fitness evaluation function is defined as follows:
  (13) $$J=\sum_{i=1}^{n}{\left(Y_{i}-Y_{i}^{\prime }\right)}^{2}/n$$
  where *Y_i_* is the actual value, $Y_{i}^{\prime }$ is the predicted value and *n* is the number of training samples.
Step 2: According to the fitness value of each particle to set the personal best position *p_best_* and global best position *g_best_*.
Step 3: Update the position *l* and velocity of each particle. Evaluate the fitness value *f*′. Then, randomly find a new position $l_{1}^{\prime }$ in the neighborhood of the particle, calculate the new fitness value ($f_{1}^{\prime }$) of the new position.
Step 4: Calculate the difference between the fitness value *f*′ and the new fitness value $f_{1}^{\prime }$, $\Delta f=f_{1}^{\prime }-f^{\prime }$.
Step 5: When $f_{1}^{\prime }\geq g_{best}$, keep the original position *l*. When Δf>0 and $f_{1}^{\prime }<g_{best}$, according the Equation (12) accept the new position $l_{1}^{\prime }$, if Δf<0 and $f_{1}^{\prime }<g_{best}$, replace the original position with the new position. Then, update the *p_best_* and *g_best_*.
Step 6: When the updates of each particle has completed, then rank all of the particles according to the each particle’s fitness value, employ the better half particles’ information to replace the other half particles’ information and update the temperature *T* = *T* × 0.9.
Step 7: If the number of iterations is equal to the maximum iterations or the *g_best_* is less than or equal to the least fitness, output the two variables of the *g_best_*; otherwise, return to Step 2.

## 4. Experiments

### 4.1. Data Description

In this paper, two cases have been considered to illustrate the effectiveness of the proposed method. The data of case 1 is a dynamic error sequence, which is derived from the measuring error of an angular instrument with anticlockwise rotation (speed 2r/min) based on standard value interpolation under room temperature, the error sequence contains a total of 240 samples. In case 2, the measuring error sequence of the length grating contains a total 334 samples. A high precision Hewlett Packard 5529A laser interferometer was used as calibration system, A-quad-B pulse from the encoder of grating length gauge is used to trigger the laser interferometer and carry out dynamic data acquisition. The software package of the HP laser interferometer is used to implement data acquisition. The speed of the target mirror is 21.1 mm/s, the experiment has been repeated five times. 

### 4.2. Preprocessing

The two datasets are both one-dimensional data, so in order to achieve the better predict results and get more information from the data, these two one-dimensional data must be converted to multi-dimensional data [26,27]. Assuming *p* is the dimension of the input vector, the reconstructed samples are listed in Table 1.

According to the reconstructed method listed in the Table 1, in case 1,the dimension number p is 16, the number of restructured sample is 224, selecting the first 124 samples for training and the final 100 samples for testing. The proportion of training samples to testing samples is 1.24:1, in case 2, the dimension *p* is 20, the number of restructured sample is 315, the first 200 samples are selected as training data and the rest are used as testing data. The proportion of training samples to testing samples is 1.73:1.

Preprocess the data by the normalized method, then perform parameter optimization and train the model.

### 4.3. Valuation Index

To further evaluate the prediction of the NAPSO-SVM model, the root mean square error (RMSE) and mean absolute percent error (MAPE) are used as evaluation indices. The definitions of MAPE and RMSE are as follows:

(14) $$\mathrm{RMSE}=\sqrt{\frac{1}{n}\sum_{i=1}^{n}{\left(Y_{i}-Y_{i}^{\prime }\right)}^{2}}$$

(15) $$\mathrm{MAPE}=\frac{1}{n}\sum_{i=1}^{n}\left|\frac{Y_{i}-Y_{i}^{\prime }}{Y_{i}}\right|$$

where *Y_i_* is the actual value, $Y_{i}^{\prime }$ is the prediction value and *n* is the number of training samples. 

The NAPSO algorithm is used to determine the punishment coefficient *C* and RBF kernel function width *σ*. The SVM model is built based on the training samples and optimal parameters. To show the performance of the proposed method, the particles swarm optimization and glowworm swarm optimization are also implemented.

### 4.4. GSO Algorithm

Glowworm swarm optimization (GSO) is a new swarm intelligence optimization algorithm, proposed by Krishnanand and Ghose [28] in 2006. GSO has some apparent differences with PSO, GSO is appropriate for multiple optima of multimodal functions. It simulates the luciferin properties of glowworms, by comparing the luciferin value to exchange information.

Like the PSO algorithm, in the GSO algorithm, each single solution is a glowworm in the search space. Each glowworm has its luciferin and neighborhood range. The value of luciferin is used to measure the quality of the solution. The value of the neighborhood range is used to determine the search scope of the glowworm. According a probabilistic mechanism, each glowworm move toward a neighbor that has a luciferin higher than its own.

Except initialization, GSO algorithm has three phases. In the first phase, each glowworm updates its luciferin, the luciferin update rule has the following equation:

(16) $$l_{i}(t+1)=(1-\rho )l_{i}(t)+\gamma J_{i}(t+1)$$

where *l_i_*(*t*) is the luciferin level for glowworm *i* at iteration *t*, *ρ* is the luciferin decay constant. *γ* is the luciferin enhancement constant and *J_i_*(*t* + *l*) is the fitness value of the objective function at glowworm *i* at iteration *t* + *l*.

In the second phase, each glowworm finds neighbors that have a luciferin level higher than its own. The set of neighbors of glowworm *i* at iteration *t* is expressed as follows:

(17) $$N_{i}(t)=\{j:d_{ij}(t)<r_{d}^{i}(t);l_{i}(t)<l_{j}(t)\}$$

where *d_ij_*(*t*) is the Euclidean distance between glowworms *i* and *j* at iteration *t*, $r_{d}^{i}\left(t\right)$ is the variable neighborhood range at glowworm *i* at iteration *t*. The probability of glowworm *t* moving toward its neighbor *j* is calculated by the following equation:

(18) $$p_{ij}(t)=\frac{l_{j}(t)-l_{i}(t)}{\sum_{k\in N_{i}(t)}l_{k}(t)-l_{i}(t)}$$

Then, the rule of the glowworm movements is given by Equation (19):

(19) $$x_{i}(t+1)=x_{i}(t)+s\left[\frac{x_{j}(t)-x_{i}(t)}{\left\|x_{j}(t)-x_{i}(t)\right\|}\right]$$

where *x_i_*(*t*) is the position of glowworm *i* at iteration *t*, $\left\|x_{j}\left(t\right)-x_{i}\left(t\right)\right\|$ is the distance between glowworms *i* and *j* at iteration *t*, s represents the step size.

In the third phase, each glowworm updates its neighborhood range by the following equation:

(20) $$r_{d}^{i}(t+1)=min\{r_{s},max\{0,r_{d}^{i}(t)+\beta (n_{t}-\left|N_{i}(t)\right|)\}\}$$

where *β* and *n_t_* are constant parameters. *r_s_* is the neighborhood range bound ($r_{d}^{i}\left(t\right)\leq r_{s}$).

## 5. Results

**Figure 1:**
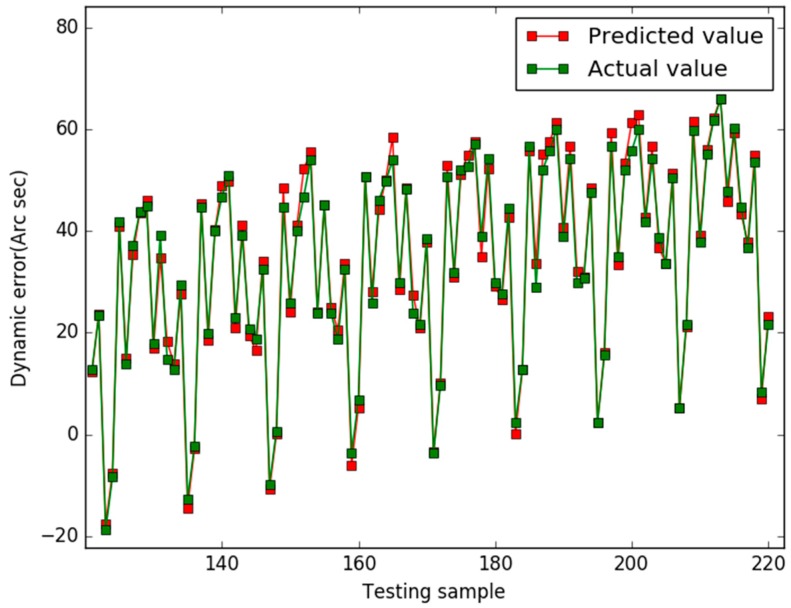
Predicted results of the NAPSO-SVM (case 1).

**Figure 2:**
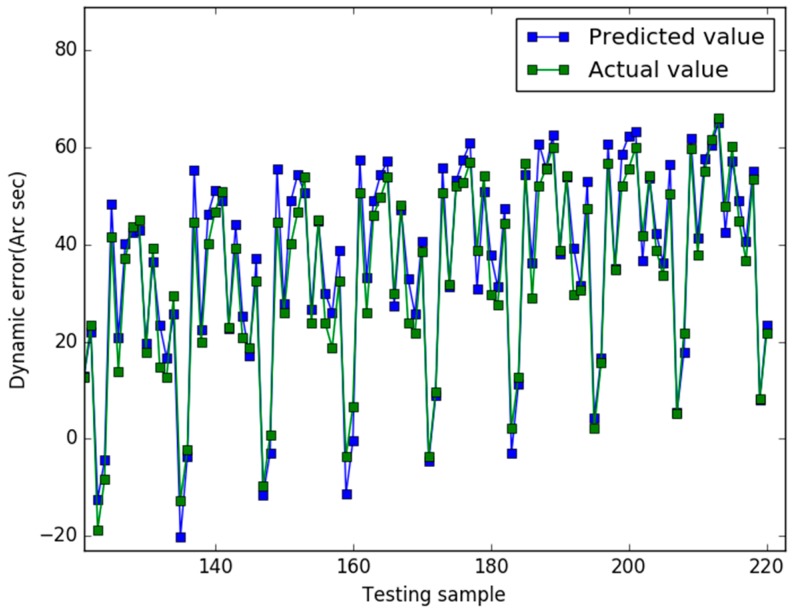
Predicted results of the PSO-SVM (case 1).

**Figure 3:**
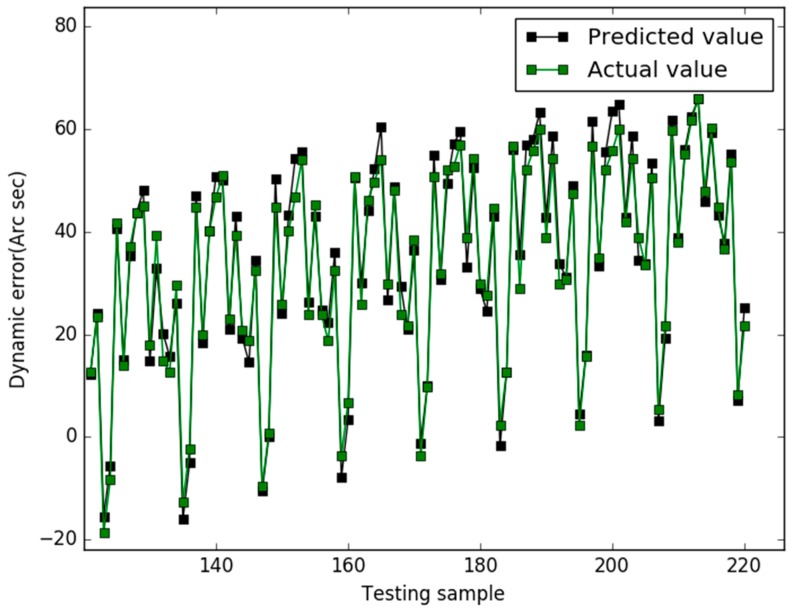
Predicted results of the GSO-SVM (case 1).

Our study used the Libsvm toolbox v3.21 (written by Chih-Jen Lin), and our algorithm are coded using Matlab 2010a and all experiments are implemented on a 4 GB RAM, 3.20 GHz Intel core i5 machine equipped with the Windows 7 operating system. In case 1, the prediction results of the three models are shown in Figure 1, Figure 2 and Figure 3, respectively. 

To make a fair comparison, the maximum generation, population size, minimum fitness value, range of gains, dimension of search space and initial positions are identical for all the algorithms. The maximum number of generations is 100, the minimum fitness value is 0.1, the size of the population is 100, and the dimension of the search space is 2. The parameters for NAPSO were set as follows: the inertia weight *w* = 0.9, the acceleration constants *c*_1_ and *c*_2_ are 2, the initial temperature is 10,000 °C, the lower limit of temperature is 1 °C, the maximum value of velocity is 1, the minimum value of velocity is −1. In GSO algorithm, the light absorption coefficient is 50, the minimum value of attractiveness is 0.8, the maximum value of attractiveness is 1.0, the value of initial step size factor is 0.5. The PSO algorithm has the same inertia weight and acceleration constant as the NAPSO algorithm. 

**Figure 4:**
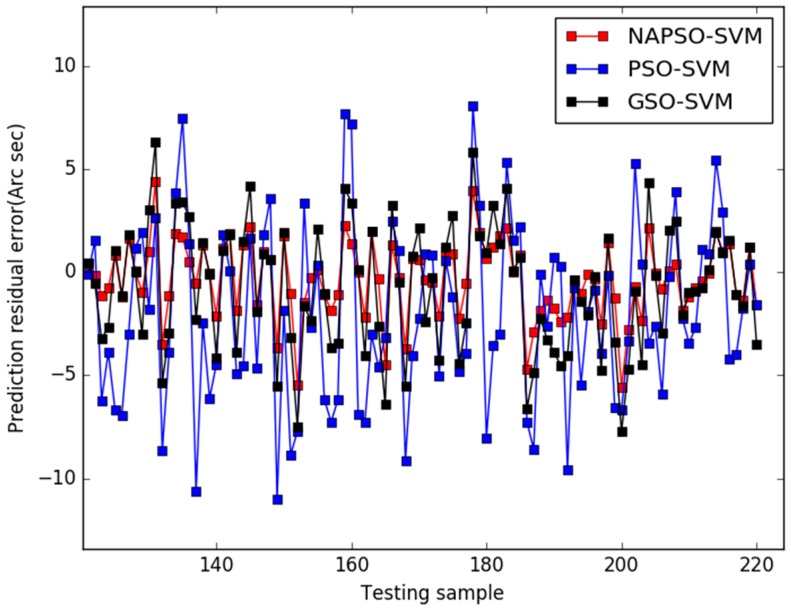
Comparison of three models for predicted residuals (case 1).

Figure 4 presents the comparison results of predicted residuals by the three models. The MAPE value and RMSE value of the three models are listed in Table 2.

In Figure 1, for the NAPSO-SVM model, the curve of predicted value is very close to the curve of the actual value. In Figure 2, the predicted value curve often lags the actual value curve. In Figure 3, the predicted value curve is close to the actual value curve, but there are many points are still outside the range of the actual curve. We could find that compared with the obvious hysteresis of the PSO-SVM and GSO-SVM models, there is a negligible hysteresis between the predicted and actual value curves with the NAPSO-SVM model, and it is closer to the actual value. We find that the NAPSO-SVM model outperforms the PSO-SVM and GSO-SVM model. The prediction performance of NAPSO-SVM is better than GSO-SVM model and accuracy much better than PSO-SVM.

The residual curves of the three models are shown in the Figure 4. The prediction residual curve of the PSO-SVM model is large, ranging from −11” to 8”, and the prediction residual of the GSO-SVM model is smaller than the PSO-SVM model. But it is still relatively large, ranging from −8” to 6”. The predicted residual of the NAPSO-SVM is smaller than the others and tends to more gentle, ranging from −5” to 4”.The results prove that dynamic measurement error prediction ability of NAPSO-SVM model is better than PSO-SVM and GSO-SVM model, and the NAPSO algorithm is an effective method for parameters optimization. To further verify the ability of the three models. Table 2 lists the comparison results between the three models for prediction accuracy indexes.

In Table 2, the MAPE value and RMSE value of the NAPSO-SVM model are smaller than those of the PSO-SVM and GSO-SVM models. The MAPE value is approximately 0.0744 for NAPSO-SVM model, compared with approximately 0.2423 and 0.1493 for the PSO-SVM and GSO-SVM models, respectively. Furthermore, the RMSE value is 0.1876 in the case of the NAPSO-SVM model. Compared with the NAPSO-SVM model, the RMSE values of the GSO-SVM model and PSO-SVM model are 0.4710 and 0.3128, respectively. In summary, the results of the Table 2 are in accord with Figure 4, and the NAPSO-SVM model has the best dynamic measurement error prediction ability among the three methods.

**Figure 5:**
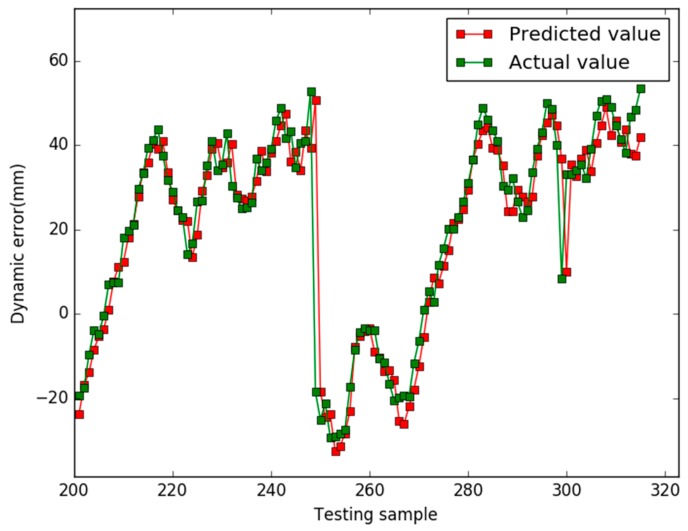
Predicted results of the NAPSO-SVM (case 2).

**Figure 6:**
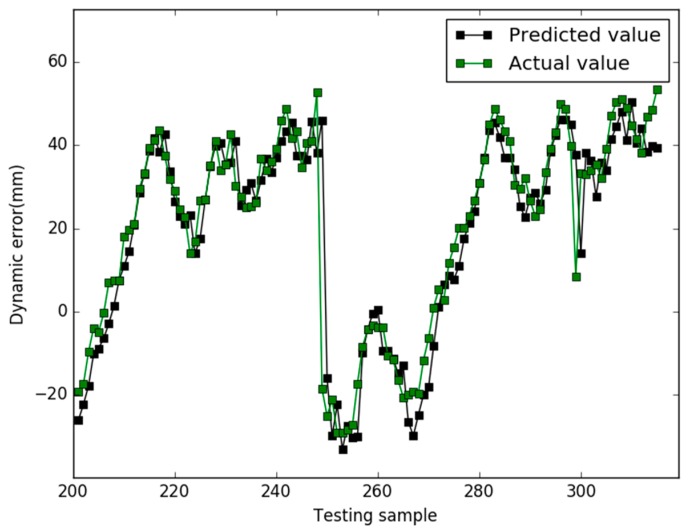
Predicted results of the PSO-SVM (case 2).

**Figure 7:**
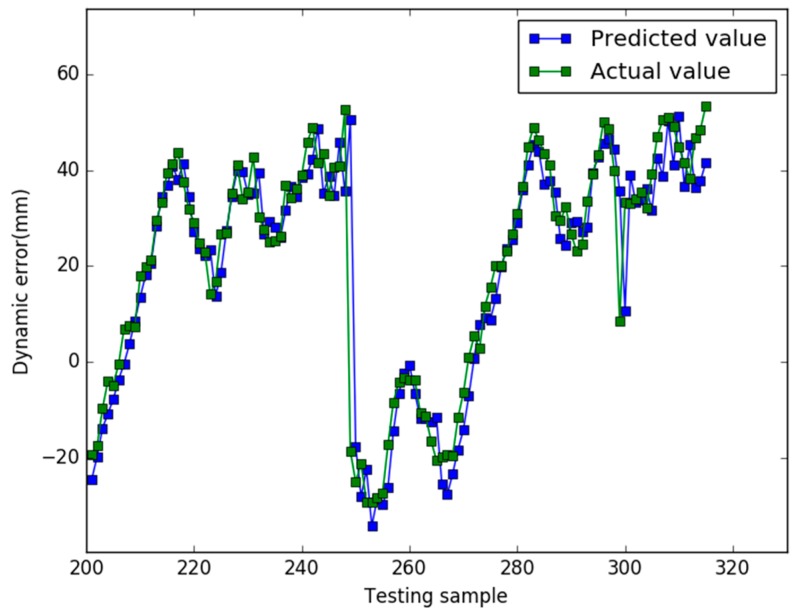
Predicted results of the GSO-SVM (case 2).

**Figure 8:**
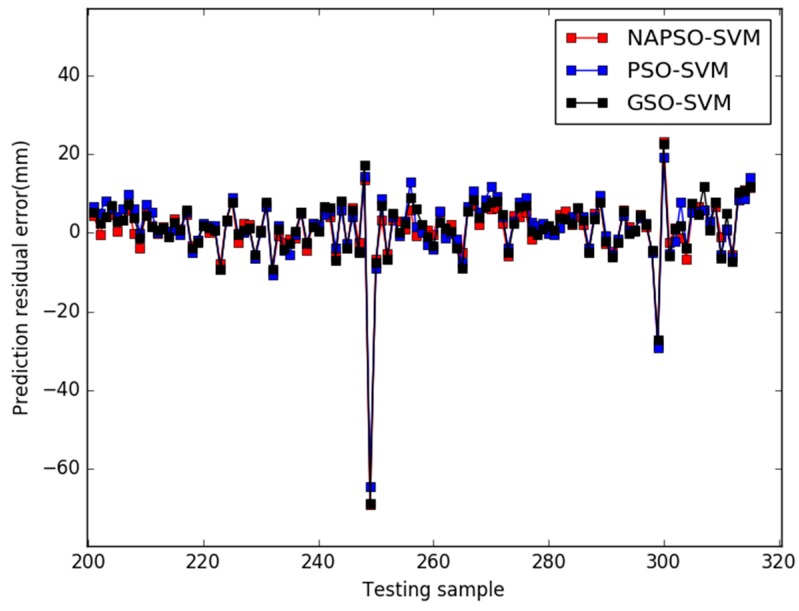
Comparison of the predicted residuals of the three models (case 2).

In case 2, the parameters of each algorithm are essentially the same as the previous case, and the prediction results of three models are shown in Figure 5, Figure 6 and Figure 7. Figure 8 shows the comparison results of the residuals predicted by the three models. The MAPE value and RMSE values of the three models are listed in the Table 3.

When the ratio of training samples and testing samples is approximately 1.73, in Figure 5, Figure 6 and Figure 7, for all models, there is hysteresis between the predicted and actual value curves on the four points (testing samples 248, 249, 299, 300). Except for the four points, the prediction curve of the NAPSO-SVM model is closest to the actual value curve, and the prediction curve of the NAPSO-SVM model is approximately the same as the actual value curve. However, unlike in case 1, the prediction results of the PSO-SVM model is nearly the same as the GSO-SVM model, but the prediction curves of these two models still lag behind the actual value curve.

In Figure 8, it is easy to see the distribution of the four hysteresis points. The prediction residual errors are very big on the four hysteresis points. Except for the four points, the prediction residual of the NAPSO-SVM model is smallest among the three models, ranging from −9.9086 to 11.3979 mm. The prediction residual of the GSO-SVM model is ranging from −9.3916 to 11.8487 mm, and the prediction residual of the PSO-SVM model is ranging from −10.7265 to 14.0340 mm. The prediction residual range of the NAPSO-SVM model is almost equal to the GSO-SVM model, and better than the PSO-SVM model.

As Table 3 shows, the MAPE value is approximately 0.3840 for the NAPSO-SVM model compared with approximately 0.5377 and 0.4403 for the PSO-SVM model and GSO-SVM model. NAPSO-SVM model has the smallest RMSE value of the three algorithms, acquiring RMSE value of 0.8015 and the RMSE values of the PSO-SVM model and GSO-SVM model are 0.8209 and 0.8356, respectively. The prediction ability of the NAPSO-SVM model is better than the other models, and GSO-SVM model has the worst RMSE value, PSO-SVM model has the worst MAPE value.

Although the data of cases is the actual data, a Gaussian noise (SNR = 15 dB) are added into the data of case 2 to prove the ability of NAPSO-SVM model, the MAPE values of the NAPSO-SVM model, PSO-SVM model and GSO-SVM model are 0.4239, 0.6035 and 0.5839, respectively. The RMSE values of the NAPSO-SVM model, PSO-SVM model and GSO-SVM model are 0.9839, 0.9868 and 0.9867. NAPSO-SVM model still has a better performance.

The results of the two cases show that the NAPSO-SVM model has the best prediction accuracy among the three methods. This indicates that the NAPSO algorithm has a better global search capability than the other two algorithms, the reason being that the updating of the position and velocity of the particles in the PSO algorithm are too dependent on the current best particle. Compared with the PSO algorithm, the NAPSO algorithm uses simulated annealing and a natural selection mechanism, so it is easier jump out of the local trap and search for the global optimal solution in the global space.

## 6. Conclusions

Dynamic measurement has been a hot area of research for several years, and dynamic measurement error prediction is a useful method to improve sensor measurement accuracy. In this study, a method of dynamic measurement error prediction based on NAPSO-optimized SVM parameters is proposed. To improve the prediction accuracy, the NAPSO algorithm is used to optimize the SVM parameters to avoid the problems of “over-fitting” and “under-fitting” of SVM. The results of the two cases show that compared with the PSO-SVM and GSO-SVM models, the NAPSO-SVM model has the better prediction accuracy. The proposed method provides a new way for predicting sensors’ dynamic measurement errors and has definite value for application in dynamic measurement. However, like the standard PSO, NAPSO has intrinsic property randomness. In the future, we plan to study which is the more effective method for improving the prediction results.

## Figures and Tables

**Table 1:** Reconstructed samples.

Input	Output
X(1),X(2),⋯,X(p)	X(p+1)
X(2),X(3),⋯,X(p+1)	X(p+2)
…	…
X(n−p),X(n−p+1),⋯,X(n−1)	X(n)

**Table 2:** Comparison of the index value among the three models (case 1).

MODEL	MAPE	RMSE
NAPSO-SVM	0.0744	0.1879
PSO-SVM	0.2423	0.4710
GSO-SVM	0.1493	0.3128

**Table 3:** Comparison of the index value among the three models (case 2).

MODEL	MAPE	RMSE
NAPSO-SVM	0.3840	0.8015
PSO-SVM	0.5377	0.8209
GSO-SVM	0.4403	0.8356

## References

[1] Cheng S., Cai Z., Li J., Gao H. (2017). Extracting Kernel Dataset from Big Sensory Data in Wireless Sensor Networks. IEEE Trans. Knowl. Data Eng..

[2] Jiang D., Li W., Lv H. (2017). An energy-efficient cooperative multicast routing in multi-hop wireless networks for smart medical applications. Neurocomputing.

[3] Jiang D., Wang Y., Han Y., Lv H. (2017). Maximum connectivity-based channel allocation algorithm in cognitive wireless networks for medical applications. Neurocomputing.

[4] Jiang D., Xu Z., Li W., Yao C., Lv Z., Li T. (2016). An energy-efficient multicast algorithm with maximum network throughput in multi-hop wireless networks. J. Commun. Netw..

[5] Yakovlev V.T. (1987). Method of Determining Dynamic Measurement Error Due to Oscillographs. Meas. Tech..

[6] Chen Y. (2015). Area-Efficient Fixed-Width Squarer with Dynamic Error-Compensation Circuit. IEEE Trans. Circuits Syst. II.

[7] Jiang D., Nie L., Lv Z., Song H. (2016). Spatio-temporal Kronecker compressive sensing for traffic matrix recovery. IEEE Access.

[8] Yakovlev V.T., Collins E., Flores K., Pershad P., Stemkovski M., Stephenson L. (2017). Statistical Error Model Comparison for Logistic Growth of Green Algae (Raphidocelis subcapitata). Appl. Math. Lett..

[9] Cheng S., Cai Z., Li J., Fang X. Drawing Dominant Dataset from Big Sensory Data in Wireless Sensor Networks. Proceedings of the 34th Annual IEEE International Conference on Computer Communications.

[10] Trapero J., Kourentzes N., Martin A. (2015). Short-term solar irradiation forecasting based on dynamic harmonic regression. Energy.

[11] Yang H., Zhang L., Zhou J., Fei Y., Peng D. (2015). Modelling of dynamic measurement error for parasitic time grating sensor based on Bayesian principle. Opt. Precis. Eng..

[12] Ge L., Zhao W., Zhao S., Zhou J. (2012). Novel error prediction method of dynamic measurement lacking information. J. Test. Eval..

[13] Jiang D., Xu Z., Lv Z. (2016). A multicast delivery approach with minimum energy consumption for wireless multi-hop networks. Telecommun. Syst..

[14] He Z., Cai Z., Cheng S., Wang X. (2015). Approximate Aggregation for Tracking Quantiles and Range Countings in Wireless Sensor Networks. Theor. Comput. Sci..

[15] Barbounis T.G., Theocharis J.B. (2007). A locally recurrent fuzzy neural network with application to the wind speed prediction using spatial correlation. Neurocomputing.

[16] Cheng S., Cai Z., Li J. (2015). Curve Query Processing in Wireless Sensor Networks. IEEE Trans. Veh. Technol..

[17] Sasikala S., Balamurugan S., Geetha S. (2016). A Novel Memetic Algorithm for Discovering Knowledge in Binary and Multi Class Predictions Based on Support Vector Machine. Appl. Soft Comput..

[18] Malvoni M., De Giorgi M.G., Congedo P.M. (2016). Photovoltaic Forecast Based on Hybrid PCA–LSSVM Using Dimensionality Reduced Data. Neurocomputing.

[19] Jiang D., Xu Z., Liu J., Zhao W. (2016). An optimization-based robust routing algorithm to energy-efficient networks for cloud computing. Telecommun. Syst..

[20] Jiang D., Zhang P., Lv Z., Song H. (2016). Energy-efficient multi-constraint routing algorithm with load balancing for smart city applications. IEEE Intern. Things J..

[21] Jiang D., Huo L., Lv Z., Song H., Qin W. (2017). A joint multi-criteria utility-based network selection approach for vehicle-to-infrastructure networking. IEEE Trans. Intell. Transp. Syst..

[22] Zhong Y., Ning J., Zhang H. (2012). Multi-agent Simulated Annealing Algorithm based on Particle Swarm Optimisation Algorithm. Int. J. Comput. Appl. Technol..

[23] Zainal N., Zain A., Radzi N. (2016). Glowworm Swarm Optimization (GSO) for optimization of machining parameters. J. Intell. Manuf..

[24] Lentka Ł., Smulko J.M., Ionescu R., Granqvist C.G., Kish L.B. (2015). Determination of gas mixture components using fluctuation enhanced sensing and the LS-SVM regression algorithm. Metrol. Meas. Syst..

[25] Kennedy J., Eberhart R. Particle Swarm Optimization. Proceedings of the First IEEE International Conference on Neural Networks.

[26] Li J., Cheng S., Cai Z., Yu J., Wang C., Li Y. (2017). Approximate Holistic Aggregation in Wireless Sensor Networks. ACM Trans. Sens. Netw..

[27] Jiang M., Luo J., Jiang D., Xiong J., Song H., Shen J. (2016). A Cuckoo Search-support Vector Machine Model for Predicting Dynamic Measurement Errors of Sensors. IEEE Access.

[28] Krishnanand K.N., Ghose D. (2009). Glowworm swarm optimization for simultaneous capture of multiple local optima of multimodal functions. Swarm Intell..

